# Wheat gene bank accessions as a source of new alleles of the powdery mildew resistance gene *Pm3*: a large scale allele mining project

**DOI:** 10.1186/1471-2229-10-88

**Published:** 2010-05-17

**Authors:** Navreet K Bhullar, Zhiqing Zhang, Thomas Wicker, Beat Keller

**Affiliations:** 1 Institute of Plant Biology, University of Zurich, Zollikerstrasse 107, 8008 Zurich, Switzerland; 2 Institute of Plant, Animal and Agroecosystem Sciences, Swiss Federal Institute of Technology, Universitätsstrasse 2, 8092 Zurich, Switzerland; 3 College of Food Science, Sichuan Agricultural University, 625014, Sichuan Yaan, China

## Abstract

**Background:**

In the last hundred years, the development of improved wheat cultivars has led to the replacement of landraces and traditional varieties by modern cultivars. This has resulted in a decline in the genetic diversity of agriculturally used wheat. However, the diversity lost in the elite material is somewhat preserved in crop gene banks. Therefore, the gene bank accessions provide the basis for genetic improvement of crops for specific traits and and represent rich sources of novel allelic variation.

**Results:**

We have undertaken large scale molecular allele mining to isolate new alleles of the powdery mildew resistance gene *Pm3 *from wheat gene bank accessions. The search for new *Pm3 *alleles was carried out on a geographically diverse set of 733 wheat accessions originating from 20 countries. *Pm3 *specific molecular tools as well as classical pathogenicity tests were used to characterize the accessions. Two new functional *Pm3 *alleles were identified out of the eight newly cloned *Pm3 *sequences. These new resistance alleles were isolated from accessions from China and Nepal. Thus, the repertoire of functional *Pm3 *alleles now includes 17 genes, making it one of the largest allelic series of plant resistance genes. The combined information on resistant and susceptible *Pm3 *sequences will allow to study molecular function and specificity of functional *Pm3 *alleles.

**Conclusions:**

This study demonstrates that molecular allele mining on geographically defined accessions is a useful strategy to rapidly characterize the diversity of gene bank accessions at a specific genetic locus of agronomical importance. The identified wheat accessions with new resistance specificities can be used for marker-assisted transfer of the *Pm3 *alleles to modern wheat lines.

## Background

Enhancing productivity in a sustainable manner is essential for future agriculture. Genetic improvement of crop plants relies on the cultivation of genotypes that possess favourable alleles/genes controlling desirable agronomic traits [1]. In the past, both early domestication and the more recent modern plant breeding have resulted in severe genetic bottlenecks, reducing the levels of genetic diversity [2]. As most of the modern genotypes cultivated today have descended from a relatively small number of landraces, the genes controlling important traits have reduced diversity compared to the gene pool of landraces and wild relatives. This is likely to make the modern varieties more vulnerable to newly emerging strains of pathogens. For example, the recent emergence of *Ug99 *stem rust race is a potential threat to wheat production worldwide [3]. Race *Ug99 *is reported to possess a unique combination of virulences that renders over 90% of worlds' wheat cultivars and breeding materials susceptible to it [3]. In view of such threats of constant genetic erosion, gene banks have been established and maintained in order to mainly preserve wild plant accessions as well as landraces [1]. Thus, gene bank collections represent very rich stocks of plant genetic diversity and can contribute significantly to the future genetic improvement of crops [4]. To use this valuable potential of genetic resources, allele mining has been suggested as an approach to identify allelic variation of relevant traits within the genetic resource collections [5,6]. This approach is best utilized for agronomically important genes with known DNA sequence, which can then be used to develop appropriate molecular tools to find new alleles [5].

Wheat is an important food crop and its production is threatened by diseases caused mostly by fungal pathogens, including powdery mildew [7,8]. Control of fungal diseases by chemicals is expensive and can have negative impacts on natural eco-systems whereas genetically based resistance offers efficient and ecologically sound control. Resistance breeding requires constant efforts to enrich the reservoir of resistance genes in wheat. More than 37 powdery mildew resistance genes have been characterized in wheat [9,10]. However, *Pm3 *is the only wheat powdery mildew resistance gene that has been cloned to date and is now known to occur in 15 functional allelic forms (*Pm3a *to *Pm3g*, *Pm3k *to *Pm3r*) [6,11-14]. The *Pm3 *gene is a distinct member of a large cluster of NBS-LRR genes on wheat chromosome 1A. The *Pm3 *alleles confer race-specific resistance to *Blumeria graminis f.sp. tritici*, the wheat powdery mildew fungus [11-13]. Eight of the 15 *Pm3 *alleles (*Pm3k *to *Pm3r*) have been recently identified [6,14] with *Pm3k *being the only allele isolated from tetraploid wheat [14] while all the other 14 functional *Pm3 *alleles were isolated from hexaploid wheat [6]. The widespread susceptible allele *Pm3CS*, which represents the consensus sequence of all the different *Pm3 *resistance alleles, has been proposed to be the ancestor of the bread wheat *Pm3 *resistance alleles [12]. The *Pm3 *alleles and their flanking sequences were found to be highly conserved forming the specific *Pm3 *haplotype [11-13]. This conservation allowed the isolation of *Pm3a *to *Pm3g *[12,13], characterized in classical breeding, as well as new alleles (*Pm3k *to *Pm3r*) which were not previously characterized by classical genetics [6,14].

The cloning of *Pm3l *to *Pm3r *[6] has been the result of an allele mining study that led to a rapid isolation of these seven new functional alleles of *Pm3*. The choice of accessions for this study was made through focused identification of germplasm strategy (FIGS) [6,15], where the eco-geographical data of powdery mildew resistant accessions was used as a reference dataset to formulate the working set of wheat landraces from environmentally similar collection sites to perform *Pm3 *allele mining. This formulated working set consisted mainly of accessions that originated from Turkey, Iran, Afghanistan, Pakistan and Armenia (96.2% of the total set screened) [6]. Thus, the accessions previously studied for *Pm3 *allelic diversity were obtained from a limited geographical region. The question remained if wheat lines originating from other regions of the world contain additional, new *Pm3 *alleles and how such new alleles would compare to the known molecular diversity. In order to further assess the diversity at the *Pm3 *locus in wheat accessions originating from geographically more diverse locations, here we established a set of 733 gene bank accessions. Thus, we have expanded the search for new *Pm3 *alleles in accessions representing 20 countries covering different continents of the world. This has led to isolation and cloning of new *Pm3 *alleles and has shed light on the molecular diversity among *Pm3 *alleles.

## Results

### Screening for powdery mildew resistant wheat accessions in gene bank material

A set of 733 wheat accessions was obtained from the gene bank of IPK, Gatersleben (Germany). These accessions were selected based on their origin from different geographical regions worldwide, complementing the earlier studied FIGS set of wheat lines which originated mostly from the Near and Middle East [6]. The 733 accessions originated from Asia, Africa, Europe, Australia and the Americas (Table 1). The entire set of these accessions was phenotypically characterized for resistance against wheat powdery mildew by screening with a set of six powdery mildew isolates. The choice of the isolates was based on their avirulence and virulence patterns to the known alleles of *Pm3*, *Pm3a *to *Pm3g*. This screening led to the identification of 154 accessions (21% of the total set) that were resistant or intermediately resistant to at least one of the six mildew isolates tested (Table 1).

**Table 1:** Summary of the selection procedure followed to isolate new *Pm3 *alleles.

Country of origin	Number of accessions	Accessions resistant or intermediately resistant to at least one powdery mildew isolate	Accessions detected with *Pm3 *haplotype	Accessions not containing any of the *Pm3a *to *Pm3g *alleles	Completely resistant candidate accessions for *Pm3 *isolation	*Pm3 *sequence obtained
**India**	92	27	21	12	7	6
**China**	90	15	8	6	2	2
**Nepal**	78	33	33	25	20	19
**Ethiopia**	63	10	8	8	4	3
**Mexico**	57	5	3	3	1	1
**USA**	53	7	5	5	2	-
**France**	51	7	3	2	2	1
**Japan**	51	1	-	-	-	-
**Russia**	38	6	3	1	-	-
**Argentina**	31	10	5	3	3	2
**Iraq**	27	7	4	4	3	2
**Canada**	25	6	5	5	3	2
**Australia**	23	7	3	2	2	1
**Tajikistan**	16	6	3	2	1	1
**Kazakhastan**	9	2	1	0	-	-
**Azerbaizan**	7	1	1	1	-	-
**Sudan**	7	2	2	2	1	1
**Switzerland**	7	1	-	-	-	-
**Kyrgyztan**	6	-	-	-	-	-
**Uzbekistan**	2	1	1	0	-	-
**Total**	733	154	109	81	51	41

The entire set of 733 accessions obtained from IPK gene bank was subjected to a hierarchical selection procedure to select for the resistant accessions which were further analysed at the molecular level for the isolation of *Pm3 *alleles.

### PCR based characterization of *Pm3 *in the resistant accessions

The 154 accessions with a resistant or intermediate resistant phenotype were subjected to molecular analysis for the *Pm3 *haplotype. First, they were screened for the presence of a *Pm3*-like gene with an STS marker obtained from haplotype studies at the *Pm3 *locus [11,13]. This STS marker amplifies a 946 bp fragment from the 5' non-coding region of *Pm3b *and is diagnostic for the presence of a *Pm3*-like gene. A total of 109 accessions out of 154 (70%) were identified with a likely presence of a *Pm3 *gene (Table 1). Subsequently, the accessions showing the presence of a *Pm3*-like gene were screened for the presence of the already known *Pm3 *alleles, *Pm3a *to *Pm3g*, with allele specific molecular markers developed previously [16]. Twenty-eight of these accessions were found to carry a known *Pm3 *resistance allele. *Pm3c *was the most frequently detected allele in this set (found in 17 accessions), followed by *Pm3b *in six accessions while *Pm3f*, *Pm3e *and *Pm3d *were detected in two, two and one accessions each, respectively. This demonstrated that the alleles of *Pm3 *in most of the tested accessions are not *Pm3a *to *Pm3g *resistance alleles and therefore, these accessions are good candidates for isolation of new *Pm3 *alleles. However, these accessions were not screened for the presence of *Pm3k *to *Pm3r*, alleles for which functional molecular markers have not yet been developed. Additionally, it cannot be ruled out that the observed resistance is caused by any of the known or still uncharacterized resistance genes present in the germplasm other than *Pm3*.

### Isolation of *Pm3 *sequences from the candidate accessions

The phenotypic and genotypic experiments described above allowed us to establish a collection of 51 candidate lines with a resistant phenotype (the intermediate resistant lines were not considered further) to specifically target for isolation of new alleles of the *Pm3 *gene (Table 1). These candidate lines were completely resistant to at least one of the isolates tested, were identified to possess a *Pm3*-like gene and lacked the known *Pm3 *alleles *Pm3a *to *Pm3g*. The *Pm3 *coding sequences were successfully amplified from 41 accessions, cloned and sequenced. In the remaining 10 accessions, amplification of a *Pm3 *sequence was not possible which might be due to absence of a coding gene or low sequence homology at the primer binding sites. Among the 41 amplified sequences, eight were identical to the susceptible *Pm3CS *[12], indicating that the observed resistance is not due to a *Pm3 *gene but is caused by other known or still uncharacterized *Pm *genes. Eighteen of these total 41 sequences were identical to the previously reported susceptible *Pm3Go*/*Jho *sequence identified from landraces of Bhutan [12]. Here, the *Pm3Go*/*Jho *allele was identified in accessions originating from India (4), Nepal (13) and China (1).

**Figure 1:**
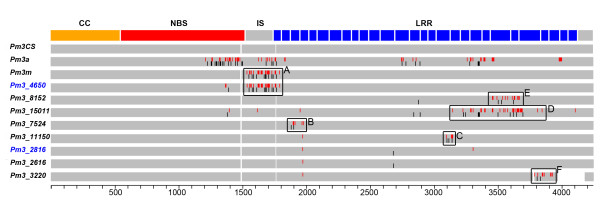
**Schematic representation of the sequence alignment (exons) of the newly identified *Pm3 *alleles**. This comparison includes the previously described *Pm3 *alleles *Pm3a*, *Pm3m *and *Pm3CS*. *Pm3CS *was the consensus as well as the reference sequence. The domains encoded by *Pm3 *alleles are depicted at the top [CC (yellow), NBS (red), Interspacer region between NBS and LRR (grey) and 28 LRRs (blue boxes)]. Red bars in the *Pm3 *alleles indicate the polymorphic nucleotides as compared to *Pm3CS *leading to non-synonymous changes in the protein. Black bars represent the polymorphic nucleotides leading to synonymous mutations. Boxes A, B, C, D, E and F indicate polymorphic sequence blocks identified in the respective alleles. The functional resistance alleles, *Pm3_4650 *and *Pm3_2816 *are labelled in blue.

The analysis of sequence diversity in the cloned genes led to the identification of eight new *Pm3 *allelic sequences, as several accessions possessed identical alleles (Figure 1 and Table 2). Four of these new *Pm3 *sequences (*Pm3_11150, Pm3_2616, Pm3_2816, Pm3_3220*) were isolated from six accessions that originated in Nepal. Three other *Pm3 *alleles were isolated from accessions from Ethiopia (*Pm3_15011*), China (*Pm3_4650*) and Argentina (*Pm3_7524*), respectively (Table 2). *Pm3_8152 *was isolated from accessions with five very different origins i.e., Sudan, Argentina, Ethiopia, Mexico and Iraq (Table 2).

**Table 2:** List and geographic origin of wheat accessions from which the eight new *Pm3 *sequences were isolated.

*Pm3 *sequence	Number of accessions carrying this allele	Accession (s)	Origin
*Pm3_11150*	2	TRI11150; TRI11152	Nepal
			
*Pm3_15011*	1	TRI15011	Ethiopia
			
*Pm3_2616*	2	TRI2599; TRI2616	Nepal
			
***Pm3_2816***	1	TRI2816	Nepal
			
***Pm3_4650***	1	TRI4650	China
			
*Pm3_7524*	1	TRI7524	Argentina
			
*Pm3_3220*	1	TRI3220	Nepal
			
*Pm3_8152*	6	TRI8152	Sudan
		TRI11477	Argentina
		TRI14797	Ethiopia
		TRI13166	Mexico
		TRI16052; TRI16081	Iraq

Some *Pm3 *alleles were identified in several accessions. The functional *Pm3 *alleles are indicated in bold.

### Newly isolated *Pm3 *alleles show overall high similarity to known *Pm3 *alleles

The eight new *Pm3 *alleles were compared to the 15 functional alleles of *Pm3 *as well as *Pm3CS*, the susceptible ancestral sequence [12]. In addition, 32 unique *Pm3 *sequences that have been previously isolated both from tetraploid (22 sequences: EU192106-25, EU192127-28,) and hexaploid wheat (10 sequences: FJ212300, FJ212303-07, FJ212309, FJ212314-15 and GU230852) were included in the comparisons [6,14]. None of these 32 alleles was found to impart resistance against the tested isolates [6,12,14].

The DNA sequence comparison of the eight new *Pm3 *sequences to the already known *Pm3 *alleles showed an overall high similarity. The *Pm3CS *sequence was used as a reference sequence in the alignment (Figure 1). The new *Pm3 *sequences also consist of two exons separated by an intron of 200 bp and encode resistance proteins with a very similar overall amino acid sequence. Six of the eight newly isolated *Pm3 *sequences are 4442 bp long (*Pm3_11150*, *Pm3_15011*, *Pm3_2616*, *Pm3_2816*, *Pm3_7524*, *Pm3_8152*), the length corresponding to that of *Pm3CS*. The *Pm3*_*4650 *sequence is 4445 bp long due to the presence of a 3 bp insertion in the region encoding LRR-1 of the gene. *Pm3*_*3220 *consisted of 4140 nucleotides and was the smallest sequence in length as it possesses a deletion spanning the last part of exon 1 and the beginning of the predicted intron. Thus, it was not possible to assign exons and intron to this sequence and it possibly represents a pseudogene. The deletion found in *Pm3*_*3220 *is identical to the one identified in the previously reported pseudogene FJ212315 [6].

The coiled coil (CC) encoding region of the new *Pm3 *sequences is completely conserved. The NBS encoding region is conserved among the new *Pm3 *alleles with the exception of two sequences (*Pm3_4650*, *Pm3_15011*). *Pm3_4650 *bears three polymorphic bases in the NBS-encoding region which are shared with *Pm3a *and *Pm3b*, while *Pm3_15011 *possesses two polymorphic bases unique to its sequence in this region. The interspacer region separating the NBS and the LRR encoding domains was also found to be conserved, except for *Pm3_4650 *and *Pm3_15011 *that possess a highly polymorphic sequence block (part of Block A, Figure 1) and a single SNP respectively, in this region. Major sequence polymorphisms between the new sequences and the *Pm3CS *consensus sequence were observed in the LRR-encoding region of the gene. These polymorphisms in the LRR region were either in the form of highly polymorphic sequence blocks or in the form of few SNPs per sequence. *Pm3_2616 *and *Pm3_2816 *differ from *Pm3CS *only by two and three SNPs, respectively, in the LRR encoding region (Figure 1). Six of the eight new sequences (*Pm3_4650, Pm3_11150, Pm3_8152, Pm3_7524 Pm3_15011 *and *Pm3_3220*) possess polymorphic sequence blocks in the LRR encoding region (part of block A and blocks B, C, D, E and F, Figure 1) in addition to the SNPs.

**Figure 2:**
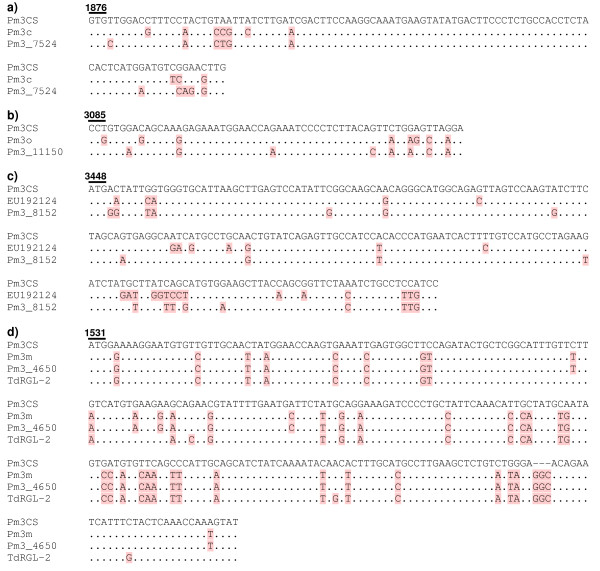
**Sequence alignment of the partially shared polymorphic nucleotide blocks among different *Pm3 *alleles**. (a) Alignment of block present in *Pm3_7524 *with *Pm3c *and *Pm3CS *(b) Alignment of the block present in *Pm3_11150 *with *Pm3o *and *Pm3CS *(c) Alignment of block present in *Pm3_8152 *with EU192124 (a *Pm3 *sequence, representative of tetraploid *Pm3 *alleles) and *Pm3CS *(d) Alignment of block present in *Pm3_4650 *with *Pm3CS *and orthologous sequence *TdRGL-2*. *Pm3CS *sequence was used as a reference sequence in all the alignments and the polymorphic nucleotides in the compared alleles are marked by pink background. Dots represent sequence identity to *Pm3CS *at the particular site. The solid line marks the triplet corresponding to the open reading frame and the numbers at the top indicate the nucleotide position in *Pm3CS*.

The polymorphic sequence block found in *Pm3_4650 *(Block A, Figure 1) is identical to the one present in *Pm3m *and *Pm3r *(Figure S1) and spans the interspacer region and the LRR-1 encoding region. In case of *Pm3_7524*, *Pm3_11150*, *Pm3_15011 *and *Pm3_8152*, the polymorphic sequence blocks are only partially shared with other previously reported alleles. The block in *Pm3_7524 *(Block B, Figure 1) consists of a total eleven nucleotide polymorphisms out of which seven are shared with *Pm3c*. Thus, *Pm3_7524 *appears to be a chimera of *Pm3CS *and *Pm3c *(Figure 2a). The *Pm3_11150 *sequence shares polymorphic residues with *Pm3o *(Figure 2b) at five out of eight polymorphic sites building the block in *Pm3_11150 *(Block C, Figure 1). The *Pm3_15011 *sequence showed high similarity with the tetraploid alleles in terms of polymorphic residues (Figure S1), especially for the presence of highly polymorphic sequence block (ranging over LRR 19 to LRR 25 of the gene (Block D, Figure 1), typically found in tetraploid alleles. In addition, *Pm3_8152 *shared 10 randomly distributed polymorphic sites out of a total of 20 sites building the block in *Pm3_8152*, with the tetraploid *Pm3 *sequences between the nucleotide positions 3450 and 3700 (Figure 2c, Figure S1). *Pm3_15011 *and *Pm3_8152 *were the only two sequences found to share polymorphic sites with the *Pm3 *alleles isolated from tetraploid wheat.

### *Pm3 *sequence diversity

The nucleotide diversity was analyzed for the coding sequence of 54 *Pm3 *sequences that have been isolated so far [present study, [6,11-14]], excluding the two pseudogene sequences (*Pm3_3220 *and FJ212315) while including *Pm3CS *and *Pm3Go/Jho*. Nucleotide diversity and ratio of non-synonymous to synonymous changes were calculated for the *Pm3 *alleles isolated from hexaploid and tetraploid wheat, for each group separately as well as together. In addition, the sequence diversity was studied specifically for the coding sequences of the distinct *Pm3 *protein domains (CC domain, NBS domain, interspacer and LRR domain, Table 3). The nucleotide diversity was found to be higher in the interspacer and LRR regions for *Pm3 *alleles isolated from both hexaploid and tetraploid wheat, compared to the diversity found in the CC and NBS domains (Table 3). In the LRR and interspacer regions, polymorphic sequence blocks of various sizes were found between the analysed *Pm3 *variants (Figure 1 and Figure S1), contributing to high values of nucleotide diversity. The highest number of non-synonymous changes were found in the LRR encoding domain, resulting in a higher value for the ratio of Ka/Ks in this region (Ka/Ks for LRR = 3.34, Table 3).

**Table 3:** Nucleotide diversity analysis and K_a_/K_s _ratios for the *Pm3 *alleles.

Domain	Total number of sites aligned	Number of polymorphic sites	Average number of polymorphic sites per 100 bp	Nucleotide diversity (π)	**Number of synonymous substitutions (K**_**s**_**)**	**Number of non-synonymous substitutions (K**_**a**_**)**	**K**_**a**_**/K**_**s **_**ratio**
**Hexaploid**							
***Pm3 *genes (excluding intron)**	4242	299	7.03	0.01008	103	252	2.44
**CC**	474	0	0.00	0.00000	0	0	-
**NBS**	1062	80	7.51	0.00742	37	47	1.27
**Interspacer**	198	37	18.4	0.0379	12	28	2.33
**LRR**	2508	182	7.25	0.0109	54	177	3.27
							
**Tetraploid**							
***Pm3 *genes (excluding intron)**	4242	185	4.36	0.01113	43	147	3.41
**CC**	474	3	0.63	0.00086	1	2	2
**NBS**	1062	4	0.37	0.00045	1	3	3
**Interspacer**	198	31	15.65	0.02598	9	22	2.44
**LRR**	2508	147	5.86	0.01643	32	120	3.75
							
**Hexaploid and tetraploid**							
***Pm3 *genes (excluding intron)**	4242	356	8.38	0.01311	121	311	2.57
**CC**	474	3	0.63	0.00038	1	2	2.00
**NBS**	1062	84	7.90	0.00451	38	50	1.31
**Interspacer**	198	43	21.39	0.03273	16	38	2.37
**LRR**	2508	226	9.02	0.01761	66	221	3.34

Nucleotide diversity was analyzed together as well as separately for *Pm3 *alleles isolated from hexaploid and tetraploid wheat, respectively. The domain positions correspond to that of consensus sequence *Pm3CS *and insertions/deletions were not counted as aligned sites. Synonymous and non-synonymous substitutions were determined by comparison of each codon in every sequence with the respective consensus sequence at that position. The number of polymorphic sites is lower than the sum of synonymous and non-synonymous substitutions because of multiple different bases in some polymorphic sites.

The average number of nucleotide differences per 100 bp was highest in the interspacer coding region. However, this high value originates from the presence of polymorphic sequence blocks in some of the hexaploid (*Pm3_4650*, *Pm3a, Pm3b, Pm3f, Pm3m *and *Pm3r*) and tetraploid (EU192116, EU192117) *Pm3 *alleles in this particular region. Comparatively low values of nucleotide diversity and Ka/Ks ratio were obtained for the CC and NBS region coding regions. This is due to very few polymorphisms found in these regions.

### Sequence exchange with *Pm3 *homologs

The entire set of 54 *Pm3 *sequences (excluding 2 pseudogenes) was compared to five *Pm3*-homologous RGAs [17] sequenced from orthologous regions of three wheat species, i.e., *T. monococcum *(*TmRGL*-1), *T. turgidum *(*TdRGL-1, TdRGL-2, TdRGL-3*) and *T. aestivum *(*TaRGL*-*9*). These are most likely paralogous genes belonging to a large cluster. These *Pm3*-like RGAs were found be very polymorphic (83.4% to 88.3% identical) as compared to the *Pm3 *alleles (>97% of sequence identity), when aligned with *Pm3CS *as a reference sequence. There were no obvious sequence blocks in the *Pm3 *alleles that would have been derived from these *Pm3*-like RGAs, except for block A (Figure 1, also mentioned above). Block A spans the interspacer and the LRR-1 encoding regions and is found specifically in *Pm3_4650*, *Pm3m *and *Pm3r*. It shares 38 out of 43 polymorphic residues with *TdRGL-2 *from *T. turgidum *(Figure 2d). The remaining five polymorphic sites at which block A and *TdRGL-2 *sequence were not identical are spread randomly over the length of the block. The presence of block A in alleles isolated from hexaploid wheat as well as in *TdRGL-2 *from *T. turgidum *indicates sequence exchange between the specific *Pm3 *allele and a paralogous RGA from the cluster.

### Functional validation of candidate *Pm3 *alleles through transient transformation

**Figure 3:**
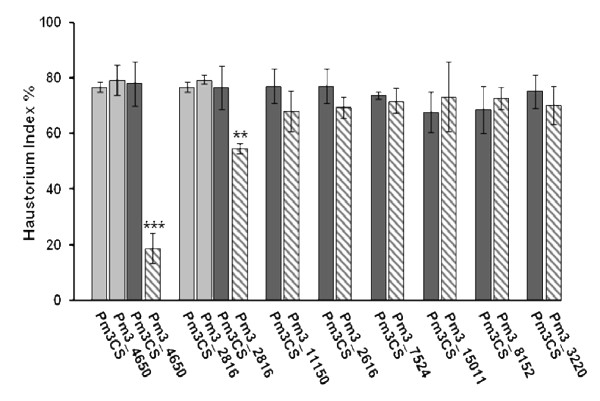
**Haustorium indices for the new *Pm3 *alleles tested in the transient transformation assay**. The eight newly identified *Pm3 *candidate alleles (bars with slanting lines) were tested with the avirulent *Bgt *isolate 98275, in comparison to the susceptible control *Pm3CS *(dark grey bars). The haustorium index (percentage of cells with haustoria) is indicated by the mean ± SD of three independent experiments, each contributing at least 50 interactions. ** Significant differences at p = 0.01; *** significant differences at p = 0.001, are also indicated. Transient assay results for the two functional alleles *Pm3*_*4650 *and *Pm3*_*2816 *in comparison to *Pm3CS *upon infection with the virulent isolate 97028 are presented by light grey bars in the graph.

The eight new *Pm3 *sequences identified in this study were tested for function in a transient transformation assay [18]. Transient transformation has previously been found to be an effective method to study powdery mildew resistance gene function in wheat [6,11,13,14]. The non-functional *Pm3CS *allele [12] was used as a control and it gave a high haustorium index (67.6 to 77.8% of susceptible interactions). In contrast, the two new alleles i.e., *Pm3_4650 *and *Pm3_ 2816 *showed a significant reduction in the haustorium index (Figure 3), with 18.7% and 54.5% average values, respectively. Transformation with the remaining six *Pm3 *allelic sequences (*Pm3*_*11150*, *Pm3_8152*, *Pm3_15011*, *Pm3_2616*, *Pm3_7524*, and *Pm3_3220*) did not result in a reduction of the haustorium index (Figure 3). To test race specific gene function of the two new alleles showing reduction in haustorium index (*Pm3_4650 *and *Pm3_ 2816*), they were also tested against the powdery mildew isolate *Bgt *97028 found to be virulent on accessions carrying these genes. In this case, no reduction of haustorium indices was observed compared to *Pm3CS *(Figure 3), demonstrating that the observed resistance activity is due to race-specificity of gene action. In conclusion, the alleles *Pm3_4650 *and *Pm3_ 2816 *are new, functionally active forms of *Pm3 *which are now called *Pm3s *and *Pm3t*, respectively. The alleles *Pm3s *(*Pm3_4650*) and *Pm3t *(*Pm3_ 2816*) were isolated from accessions that originated from China and Nepal, respectively. The repertoire of functional *Pm3 *alleles now includes 17 genes, making it one of the largest allelic series of plant resistance genes.

## Discussion

### Gene bank accessions as source of genetic diversity for the *Pm3 *gene

Genetic diversity is highly relevant for improvement of crop traits by breeding. The widespread use of genetically uniform varieties provides an ideal genetic environment for disease epidemics; for example, the devastating 1970 epidemic of corn leaf blight caused by the fungal pathogen *Bipolaris maydis *happened due to widespread deployment of genetically uniform varieties [4]. Crop genetic resources harbour a treasure of undiscovered allelic variants and thus provide an opportunity for genetic improvement of cultivated species. In the past, a large number of agronomically important genes, including disease resistance genes, were introgressed from wild relatives and landraces into the cultivated species. For example, the two very important wheat genes *Rht1 *and *Rht2 *providing the foundation for green revolution were introgressed from cultivar 'Norin10' that originally inherited these genes from Japanese landrace "Shiro Daruma" [4].

Previously, the approach to utilize gene bank accessions was mostly restricted to phenotyping for a particular trait and then introducing it to elite cultivars through repeated back-crossing. More recently, new approaches based on molecular analysis have emerged as a promising alternative. These approaches include linkage mapping analysis [2,19], association mapping [20-22] and allele mining [6]. Recently, seven new functional alleles of powdery mildew resistance gene *Pm3 *(*Pm3l *to *Pm3r*) have been isolated from hexaploid wheat landraces following the allele mining approach [6]. The focused identification of germplasm strategy used in this study has been successful in identifying new sources of powdery mildew resistance. However, the source of germplasm in this set was somewhat limited and the diversity of *Pm3 *alleles remained unassessed in accessions that originated from countries other than the ones selected through FIGS. In this study here, we have isolated new *Pm3 *alleles from Nepal, Ethiopia, China, Argentina, Sudan, Mexico and Iran. The newly identified functional *Pm3 *alleles in the present study, *Pm3s *and *Pm3t*, originated from China and Nepal respectively. This demonstrates that functional *Pm3 *genetic diversity, i.e. new resistance genes, has not been exhausted yet and Asian germplasm accessions are candidates to further screen for unique powdery mildew resistance sources. These new findings will also be useful in an iterative process to further refine the FIGS selection process by taking the data of newly identified resistant accessions into consideration, when searching for more variation in powdery mildew resistance.

### *Pm3*, a gene with a large number of functional alleles

*Pm3 *is one of the few plant resistance genes with a high number of known functional alleles. For the *Mla *locus of barley, more than 30 race-specific resistance alleles have been described [23-25]. The sequence comparison of six isolated *Mla *resistance alleles (*Mla1, Mla6, Mla7, Mla10, Mla12 *and *Mla13*) revealed 90% sequence identity which led to the hypothesis that the *Mla *powdery mildew resistance specificities represent allelic variants [23,24,26-28]. This hypothesis was further supported by a recent study, where 23 additional candidate *Mla *cDNAs have been isolated and they also represented allelic variants of the previously described *Mla *resistance alleles [25]. Forty-seven alleles of the *RPP13 *gene of *A. thaliana *controlling resistance to downy mildew have been isolated in two studies (24 and 23 alleles each), where most of the accessions were collected from natural populations of *A. thaliana *across the United Kingdom [29,30]. The flax rust resistance locus *L *is another example of a plant resistance gene with a large number of alleles and has been described to occur in 13 functional forms [31]. The two functional *Pm3 *alleles reported in this study (*Pm3s *and *Pm3t*) further extend the previously known *Pm3 *allelic series (*Pm3a *to *Pm3g*, *Pm3k *to *Pm3r*) [6,12-14]. In addition to the 17 functional alleles, several *Pm3 *alleles were isolated [present work, [6,14]] from hexaploid (16 sequences) and tetraploid wheat accessions (22 sequences), for which no function as resistance genes could be assigned. Thus, a total of 56 naturally occurring *Pm3 *sequences are now known, including the susceptible ancestral sequence *Pm3CS *(found in both tetraploid and hexaploid wheat species).

### *Pm3 *allelic diversity: a molecular insight

The overall nucleotide diversity for all the *Pm3 *sequences was calculated to be lower (π = 0.01) than that described for other allelic series of plant resistance genes, for example, the *RPP13 *alleles (π = 0.045) in *A. thaliana*, the *Mla *powdery mildew resistance alleles (π = 0.043) in barley and *L *alleles (π = 0.033) conferring rust resistance in flax [23,25,29,31]. These data support the hypothesis of a recent divergence for *Pm3 *alleles, as previously proposed [12]. Most of the sequence polymorphisms were found in the LRR domain. The high non-synonymous to synonymous nucleotide divergence value calculated for the LRR domain (Ka/Ks = 3.34) indicates a strong diversifying selection acting on this part of the gene. This data support a major role of the LRR domain in recognition specificity for *Pm3*-mediated resistance in wheat. This corresponds well to other studies where sequence variability in resistance genes or alleles was mostly found in the LRR encoding regions [31,32] and diversifying selection was detected in solvent exposed residues [33]. The LRR encoding domain of the barley *Mla *gene was also found to exhibit highest nucleotide diversity with a π value of 0.074 [25].

The *Pm3 *sequences differed mostly by point mutations and/or polymorphic sequence blocks that were further reshuffled between alleles. For example, the newly isolated allele *Pm3_4650 *(this work) possesses a sequence polymorphic block (block A) identical to that present in *Pm3m *and *Pm3r *[6]. *Pm3_7524 *and *Pm3_11150 *(this work) share sequence identity within the polymorphic block with *Pm3c *and *Pm3o*, respectively, however only partially. Previously, it has been shown that *Pm3r *shares such a polymorphic sequence block with *Pm3a *while *Pm3o *shared another unique polymorphic block with FJ212300, FJ212307 and FJ212308 [6]. *Pm3l*, *Pm3p*, *Pm3q *and FJ212314 shared polymorphic blocks among each other, either completely or partially [6]. These polymorphic sequence blocks possibly derive from gene conversion and/or recombination events among different *Pm3 *alleles. Sequence exchange by gene conversion and recombination has been reported to be one of the major mechanisms of resistance gene evolution [34,35]. It is also likely to be a main mechanism of *Pm3 *evolution in tetraploid and hexaploid wheat.

Interestingly, the *Pm3 *alleles from hexaploid and tetraploid wheat form two distinct groups specifically with regard to polymorphic sequence blocks unique to their respective ploidy levels. This was evident from the presence of a single large polymorphic sequence block covering LRR19 to LRR25 [14], typically found in *Pm3 *sequences isolated from tetraploid wheat accessions (found in 16 out of 23 tetraploid *Pm3 *sequences), with the rest of polymorphisms mostly in the form of SNPs (see Additional file 1). The remaining seven tetraploid *Pm3 *sequences (out of 23) either partially shared this block (EU192198), had an entirely different sequence polymorphism pattern (*Pm3k*, EU192119) or did not have the block but only a few SNPs (EU192121, EU192122, EU192123, EU192127) in comparison to *Pm3CS*. The *Pm3 *alleles isolated from hexaploid wheat accessions did not possess this particular polymorphic sequence block, except for *Pm3_15011 *and *Pm3_8152 *that completely or partially shared it. Different hexaploid *Pm3 *alleles possessed several small polymorphic blocks scattered over the length of the gene (with a majority in the LRR encoding region), in contrast to one large and evident block in tetraploid alleles.

Some of the *Pm3 *sequences appear to have originated due to insertion and deletion events, in addition to the point mutations and gene conversion [this work, [6,11-13]]. For example, *Pm3_4650*, *Pm3a*, *Pm3b*, *Pm3f*, *Pm3m *and *Pm3r *bear a single 3bp insertion in comparison to *Pm3CS *in the LRR-1 encoding region of the gene, and in addition to this, *Pm3a *and *Pm3b *bear another triplet insertion in the NBS encoding part of the gene. *Pm3l*, *FJ212303 *and *FJ212314 *possess 45 bp deletions in the LRR encoding region, while FJ212305 lack 3bp in the NBS encoding region of the gene. These InDel mutations do not compromise the full-length open reading frames, except for the two pseudogenes FJ212315 and *Pm3_3220*. The *Mla *specificities, *Mla1*, *Mla6 *and *Mla13 *were also shown to possess insertions or deletions that mostly occurred in multiples of three nucleotides [36]. It was also suggested previously that *Pm3 *recognition specificities can be generated rapidly by few mutational events in the *Pm3CS *sequence [12]. This was evident with the isolation of functional *Pm3 *alleles that differ from *Pm3CS *by a few nucleotides. The new allele *Pm3_2816 *differs by three nucleotides from the *Pm3CS *sequence.

## Conclusions

Strategies that have been undertaken to approach genetic resources in order to identify new agronomically important genes include random selection based on geographical origin of accessions, core collections and focused identification of germplasm strategy (FIGS). In different independent studies, these strategies have yielded new alleles/genes of high importance. However, efforts are still required to further improve these strategies so as they can potentially reduce phenotypic or genotypic screening but allow effective output. The wild accessions and landraces available in the gene banks evolved under a variety of edaphic and climatic environments and this might have resulted in evolution of ecotypes adapted to specific local environments. Therefore, the choice for subset of accessions which is economically feasible to screen can be based on previous successful reports. For example, in the particular case of *Pm3 *alleles, the previously unknown functional alleles have been isolated from accessions with their origin in Turkey, Afghanistan, Turkmenistan, China and Nepal [this work, [6]] out of over 30 countries tested. Therefore, it seems a promising approach to identify more powdery mildew resistance sources specifically from accessions originating from these countries and future projects could be focused on such origins.

The new functional alleles isolated in this project can now be transferred to susceptible but economically important wheat varieties as single genes or R-gene cassettes. Besides this more applied aspects in wheat breeding, the now available 56 natural genetic variants of the *Pm3 *gene isolated from accessions from all over the world provide a unique experimental set to explore and understand the molecular basis of the allelic specificity of resistance genes. The high conservation of sequence among *Pm3 *alleles showing resistance against different spectra of pathogen races indicate that the *Pm3 *specific resistance function must be determined by a relatively small number of nucleotides that are polymorphic among these alleles.

## Methods

### Seed material

The set of 733 wheat gene bank accessions used in the study was obtained from Dr. Andreas Börner and Dr. Andreas Graner, Leibniz Institute of Plant Genetics and Crop Plant Research IPK, Gatersleben, Germany. The accessions of this set were chosen on the basis of their geographical origin and were from India (92 accessions), China (90 accessions), Nepal (78 accessions), Ethiopia (63 accessions), Mexico (57 accessions), USA (53 accessions), France (51 accessions), Japan (51 accessions), Russia (38 accessions), Argentina (31 accessions), Iraq (27 accessions), Canada (25 accessions), Australia (23 accessions), Tajikistan (16 accessions), Kazakhastan (nine accessions), Azerbaijan (seven accessions), Sudan (seven accessions), Switzerland (seven accessions), Kyrgystan (six accessions) and Uzbekistan (two accessions).

### Phenotypic characterisation of the wheat accessions and powdery mildew isolates

In order to select accessions resistant to powdery mildew, detached leaf segments from seven day old plants were placed on phytagar media and were subjected to infection with powdery mildew isolates. The six powdery mildew isolates used in the study *Bgt *98275, *Bgt *Syros 2000.15, *Bgt *96224, *Bgt *97011, *Bgt *96229 and *Bgt *DB-Asosan were selected on the basis of their virulence and avirulence reactions [6] on the lines carrying *Pm3 *alleles, *Pm3a *to *Pm3g*. The mildew isolates were maintained on wheat cv. Kanzler with weekly transfer to fresh plants/leaf segments. The isolates used in the study were obtained either from the former mildew collections of Agroscope Reckenholz-Tänikon (ART), Zürich, Switzerland, Institut National de Recherche Agronomique INRA, Rennes, France or our own powdery mildew collection at University of Zürich, Switzerland. The scoring was done 9-10 days after infection [5]. The phenotypes were classified into three categories: resistant (R), intermediate [(I) with two further categories: Intermediate resistant (IR) and intermediate susceptible (IS)] and susceptible lines (S).

### Isolation of new *Pm3 *alleles

Alleles were amplified by using *Pm3*-locus specific, long range PCR amplification followed by a nested long range PCR. PCR primers were based on the upstream and downstream sequence of the coding region of the *Pm3b *allele. PCR amplification of the *Pm3 *alleles was carried out with the Herculase-II fusion high-fidelity DNA polymerase. Amplified fragments were cloned into the multiple cloning site of expression vector PGY1 [18] between a 540 bp fragment of the 35SCaMV promoter and the 35SCaMV terminator. DNA sequencing was performed with an Applied Biosystems Capillary Sequencer model 3730. The obtained sequences were confirmed by independent amplification and sequencing.

### Sequence analysis

Sequence assembly was performed using the Gap4 program of the Staden Package http://staden.sourceforge.net/. The ClustalX software [37] was used for sequence alignments which were further analysed in the program Genedoc http://www.nrbsc.org/gfx/genedoc/index.html. For sequence diversity analysis, multiple sequence alignments were performed with CLUSTALW [38] using default gap creation and gap extension penalty. Nucleotide diversity and Ka/Ks ratios were calculated with program DnaSP http://www.ub.es/dnasp.

### Single cell transient transformation assay and microscopy

The transient gene expression assay [6,11,12,18] is based on transformation of single leaf epidermal cells, followed by subsequent inoculation of transformed leaves with specific powdery mildew isolates. Biolistic bombardment was performed as described in Yahiaoui *et al *[12]. Leaves of the powdery mildew susceptible line Chancellor were bombarded with a 1:1 (wt/wt) mixture of pUbiGUS containing the GUS reporter and the PGY1 vector containing the *Pm3CS *gene or the newly isolated *Pm3 *alleles. Leaf segments were infected with appropriate powdery mildew isolate, four hours after the bombardment. All the eight new *Pm3 *sequences were tested against avirulent isolate Bgt 98275 and the two functional *Pm3 *alleles (*Pm3_4650 *and *Pm3_2816*) were also tested against virulent isolate 97028. Staining for GUS activity was carried out 44 hours post inoculation. After 18 hours of GUS staining, the fungal structures were subsequently stained with Coomassie blue. GUS expressing epidermal cells attacked by a single germinating spore were evaluated by transmission light microscopy. A susceptible interaction was characterized by a mature haustorium and elongating secondary hyphae whereas a resistant interaction was characterized by the presence of an appressorium. At least three independent experiments were carried out, each time counting at least 50 interactions.

### Accession numbers

*Pm3_7524*: GU230853, *Pm3_4650*: GU230854, *Pm3_2616*: GU230855, *Pm3_15011*: GU230856, *Pm3_2816*: GU230857, *Pm3_11150*: GU230858, *Pm3_8152*: GU230859, *Pm3_3220*: GU230860, *Pm3Go/Jho*: GU230852

## List of abbreviations

SNPs: Single nucleotide polymorphisms; CC: coiled coil; NBS: Nucleotide binding site; LRR: Leucine rich repeats, InDels: Insertions and deletions

## Authors' contributions

NKB contributed by designing the project, conduct of research, data analysis and by writing this paper, ZZ contributed in the conduct of research, TW contributed in sequence diversity analysis, BK contributed in design of study, data analysis and by writing this paper. All authors read and approved the final manuscript.

## Supplementary Material

Additional file 1 **Schematic representation of the *Pm3 *sequence alignment (exons) of the 54 *Pm3 *alleles (excluding two pseudogenes)**. Schematic representation of the *Pm3 *sequence alignment (exons) of the 54 *Pm3 *alleles (excluding two pseudogenes). *Pm3CS *was used as a reference sequence. The domains encoded by *Pm3 *alleles are depicted at the top [CC (yellow), NBS (red), Interspacer (grey) and 28LRRs (blue boxes)]. The EU192106 to EU192128 and *Pm3k *are the *Pm3 *alleles isolated from tetraploid wheat. The sequences *Pm3a *to *Pm3g*, *Pm3l *to *Pm3r*, FJ212300 to FJ 212315, and the newly isolated *Pm3 *sequences in this study (*Pm3_4650*, *Pm3_8152*, *Pm3_15011*, *Pm3_7524*, *Pm3_11150*, *Pm3_2816*, *Pm3_2616*) were isolated from hexaploid wheat accessions. Click here for file
