# Supplementary figures and images for: Wheat gene bank accessions as a source of new alleles of the powdery mildew resistance gene Pm3: a large scale allele mining project
Source: BMC Plant Biol. 2010 May 17;10:88. doi: 10.1186/1471-2229-10-88 (PMC3095356; doi:10.1186/1471-2229-10-88)

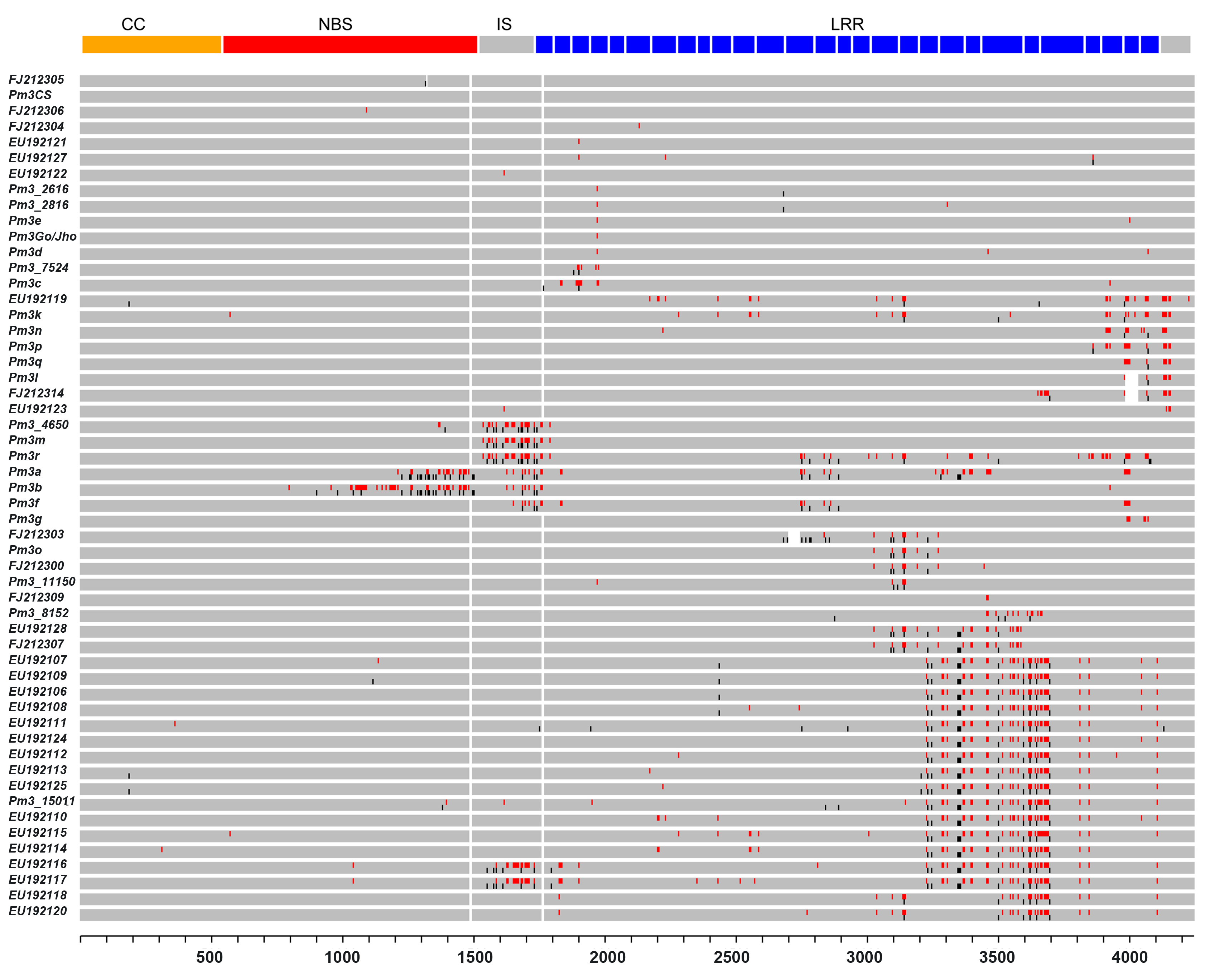

Supplement: Additional file 1 — Schematic representation of the Pm3 sequence alignment (exons) of the 54 Pm3 alleles (excluding two pseudogenes). Schematic representation of the Pm3 sequence alignment (exons) of the 54 Pm3 alleles (excluding two pseudogenes). Pm3CS was used as a reference sequence. The domains encoded by Pm3 alleles are depicted at the top [CC (yellow), NBS (red), Interspacer (grey) and 28LRRs (blue boxes)]. The EU192106 to EU192128 and Pm3k are the Pm3 alleles isolated from tetraploid wheat. The sequences Pm3a to Pm3g, Pm3l to Pm3r, FJ212300 to FJ 212315, and the newly isolated Pm3 sequences in this study (Pm3_4650, Pm3_8152, Pm3_15011, Pm3_7524, Pm3_11150, Pm3_2816, Pm3_2616) were isolated from hexaploid wheat accessions. [file 1471-2229-10-88-S1.TIFF]
